# Effects of Luteolin Treatment on Postharvest Quality and Antioxidant Capacity of Nanfeng Tangerines

**DOI:** 10.3390/foods14010068

**Published:** 2024-12-29

**Authors:** Wenjuan Dong, Xiaohan Wang, Miaolian Xiang, Jinyin Chen, Jiaoke Zeng, Ming Chen

**Affiliations:** Jiangxi Provincial Key Laboratory for Postharvest Storage and Preservation of Fruit and Vegetables, College of Agronomy, Jiangxi Agricultural University, Nanchang 330045, China; 19958564153@163.com (W.D.); xhan568@163.com (X.W.); mlxiang2010@126.com (M.X.); jinyinchen@126.com (J.C.)

**Keywords:** luteolin, Nanfeng tangerine, postharvest quality, antioxidant capacity

## Abstract

Postharvest quality deterioration is a major factor affecting the economic value and marketing of Nanfeng tangerines. The objective of this study was to explore the effects of luteolin treatment on the postharvest quality and antioxidant capacity of Nanfeng tangerines. We applied 1 g/L and 3 g/L luteolin to fruit after harvest and evaluated the decay rate, postharvest quality, and antioxidant capacity during a 60-day storage period at room temperature. The results indicated that, compared to untreated fruit, Nanfeng tangerines treated with 3 g/L luteolin exhibited enhanced appearance and flavor quality, as well as delayed disease incidence, during room-temperature storage. Additionally, flavor quality analysis revealed that luteolin treatment maintained high levels of titratable acid (TA) by delaying the degradation of organic acids such as citric, tartaric, succinic, ascorbic, and oxalic acids. Furthermore, luteolin treatment inhibited malondialdehyde (MDA) and H_2_O_2_ accumulation by enhancing the content of total phenols and flavonoids content, augmenting antioxidant enzyme activities (peroxidase (POD), catalase (CAT), and superoxide dismutase (SOD)), and elevating the overall antioxidant capacity measured through the 2,2-diphenyl-1-picrylhydrazyl (DPPH) free radical scavenging rate. Collectively, these results demonstrate that luteolin has potential as a preservative for promoting postharvest quality and antioxidant capacity. Additionally, our findings elucidate the mechanisms by which plant-derived flavonoids contribute to the preservation of freshness.

## 1. Introduction

Nanfeng tangerines (*Citrus reticulata* Blanco.) are a unique fruit cultivated in Jiangxi Province, China [1,2]. As a fruit with over 1300 years of cultivation history, Nanfeng tangerines are highly favored by consumers owing to their golden color, delightful taste, and notable health beneficial compounds (e.g., amino acids, selenium, and vitamins) [3]. Fruits are harvested at the commercial maturity stage (indicated by solid-acid ratio) and have a prolonged shelf life under suitable storage conditions [3]. However, after harvest, Nanfeng tangerines are prone to both mechanical damage and fungal attacks, leading to a decline in and loss of quality due to decay during storage [4,5]. Quality deterioration of citrus fruit is partly attributed to the imbalance of reactive oxygen species (ROS) and antioxidants. Over accumulation of H_2_O_2_ and MDA content may led to membrane-lipid peroxidation, while the ROS scavenging system, including antioxidant enzymes and non-enzymatic components, are enhanced to alleviate oxidative injuries or disease [6]. Therefore, a variety of approaches have been developed to maintain Nanfeng tangerine postharvest quality and extend the shelf life, including preharvest treatment [7], postharvest hot water dipping treatment [8], coating [9], fruit extraction [10], naringin application [11], and antagonistic microorganisms [12]. Although various treatments have been explored for citrus preservation, preventing postharvest storage loss is mainly dependent on chemical fungicides [13]. However, the use of chemical fungicides in citrus postharvest processing is limited by the existence of unexpected residues and their possible consequences on human health and environmental pollution [14,15]. Hence, the development of safer and more efficient postharvest handling methods is crucial for preserving postharvest quality.

Luteolin is a plant-derived flavone compound that is generally found in fruits and vegetables like tomatoes, capsicum, celery, and dragon fruit [16,17]. With its broad spectrum of bioactivities, such as antioxidant, anti-inflammatory, and anti-cancer properties, it has been traditionally utilized in Chinese medicine for its potential in promoting health [18,19]. In recent years, luteolin has been reported to play multifunctional roles in postharvest fruit color changes, quality maintenance, and disease resistance. Tang et al. [20] demonstrated that luteolin inhibits reddening in winter jujubes by limiting anthocyanin synthesis and chlorophyll degradation, along with increasing the antioxidant capacity and alleviating browning. Liu et al. [21] revealed that luteolin maintains sweet cherry postharvest quality and enhances disease resistance by activating phenylpropanoid metabolic pathways. However, the effects and physiological mechanisms of luteolin on postharvest citrus fruit quality require further investigation.

In this study, our objective was to assess the impact of different concentrations of luteolin on the storage quality of Nanfeng tangerines. To this end, assessment of non-enzymatic antioxidants and primary antioxidant enzymes was conducted subsequent to luteolin treatment. Our results provide insights into quality maintenance and disease resistance in citrus fruits through the utilization of luteolin and other flavonoids.

## 2. Materials and Methods

### 2.1. Plant Materials and Treatments

**Figure 1:**
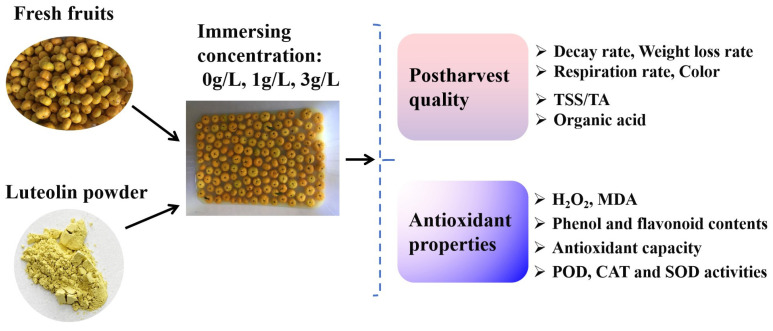
A flow diagram depicting the materials and treatments.

Nanfeng tangerines (*Citrus reticulata* Blanco) were picked in 2022 from Nanfeng County, Jiangxi Province, China, at the stage of commercial maturity. Fruits with identical color and size (Single fruit weight of 19–22 g, CCI value of 2.0–4.0), without any imperfections, were collected and promptly transported to the laboratory on the day of harvest. The fruit was transported using a logistics vehicle under ambient temperature conditions, with fruit samples randomly split into three groups of about 600 fruits each. The fruits in each group underwent a 2-min soaking in either tap water (CK, control), 1 g/L luteolin (L1U; McLean Biochemical Technology Co., Ltd., Shanghai, China), or 3 g/L luteolin (L3U). Luteolin was prepared with 10% (*v*/*v*) absolute ethanol, subsequently added to 50 °C tap water to the final concentration described above. After immersion, the fruits underwent air-drying, and were then subsequently packed into 0.03-mm thick polyethylene film and placed in storage at room temperature (with 85–90% relative humidity) for 60 days. Fruit decay rate, weight loss rate, respiration rate, flavor quality, and antioxidant capacity were determined at 10-day intervals throughout the storage period (Figure 1). Peel and pulp tissues from each sampling point were ground individually in liquid nitrogen, and the samples were stored at −80 °C for further analyses.

### 2.2. Evaluation of Decay Rate and Weight Loss Rate

Four biological replicates of 100 fruits each were prepared to evaluate the decay rate. The decay rate was represented by the percentage of fruit that was diseased [10]. Thirty fruits were utilized to calculate the rate of weight loss. Weight loss was quantified as a percentage (%) of the initial weight and was calculated every 10 d [10].

### 2.3. Measurement of Peel Color

At each sampling point, three replicates, each containing ten fruits, were randomly chosen for assessing fruit quality and physiological parameters at room temperature. Changes in fruit peel color values for L*, a*, and b* were determined with a colorimeter (CR-400; Konica Minolta, Inc., Tokyo, Japan). The color value of each fruit was averaged from four measurement sites at the fruit equator. The citrus color index (CCI) was calculated by the formula: 1000 × a*/(L* × b*).

### 2.4. Measurement of Respiration Rate

Using a respiration-measuring meter (JFQ-3150H; Jun-Fang-Li-Hua Tech., Beijing, China), the respiration rate of fruits was measured. Ten fruits from each replicate were sealed within a chamber, and changes in CO_2_ levels within 5 min were recorded at room temperature. The respiration rate was quantified as mg CO_2_/kg fresh weight (FW)/h.

### 2.5. Measurement of Total Soluble Solids, Titratable Acid, and Solid/Acid Ratio

The content of total soluble solids (TSS, %) was analyzed utilizing a refractometer (PAL-1; Aiago, Tokyo, Japan). Titratable acid (TA, %) was measured by titrating Nanfeng tangerine juice with sodium hydroxide, and the results were presented as the % of citric acid [10]. All measurements were carried out three times. The ratio of TSS to TA content was used to calculate the solid-to-acid ratio (TSS/TA, %).

### 2.6. Measurement of Organic Acid Component

The extraction of organic acids was carried out following the procedure detailed by Gao et al. [22] with minor modifications. To a 4 mL solution of 80% ethanol, 2 g of frozen pulp powder was added. The homogenate was incubated at 35 °C in a water bath for 20 min, followed by centrifugation at 4000 × rpm for 20 min at room temperature. After undergoing three centrifugation cycles, the supernatant was collected and supplemented with 80% ethanol to achieve a final volume of 14 mL. Then, 1 mL of supernatant underwent drying through rotary evaporation, was redissolved in ultrapure water, and then filtered using a water syringe filter (0.44 μm aperture, 13 mm diameter). A high-performance liquid chromatograph (LC-2030 Plus; Shimadzu, Kyoto, Japan) equipped with a C18-AQ column (4.6 mm × 250 mm; Shimadzu, Kyoto, Japan) and a Shimadzu diode array detector (SPD-M20A) was used to measure the organic acids. The mobile phase consisted of 0.1 M phosphate buffer (pH 2.7) flowing at a rate of 1 mL/min with an injection volume of 10 μL. Detection of organic acid components were conducted at 210 nm and expressed as mg/g. These measurements were repeated thrice.

### 2.7. Measurement of Fruit Peel H_2_O_2_ and Malondialdehyde Content

The H_2_O_2_ content was measured using the procedure outlined by Patterson et al. [23] with minor changes. In 2 mL of ice-cold acetone, frozen fruit peel powder (0.2 g) was homogenized. After centrifugation at 12,000× *g* for 20 min at 4 °C, 1 mL of supernatant was obtained. It was then combined with 0.1 mL of a 10% titanium sulfate–hydrochloric acid solution, 0.2 mL of concentrated ammonia, and 3 mL of 2 M sulfuric acid. Absorbance was recorded at a wavelength of 415 nm. These measurements were repeated thrice. The H_2_O_2_ content was calculated as follows:

$$H_{2}O_{2}\;\mathrm{content}\;(\mathrm{mmol}/g\;\mathrm{FW})=\frac{n\times V}{Vs\times m}$$

where *n* is the amount of substance obtained from the standard curve (mmol), *V* is the volume of extraction buffer (mL), *Vs* is the volume of supernatant used for H_2_O_2_ detection (mL), and *m* is the mass of the Nanfeng tangerines (g).

Malondialdehyde (MDA) was measured following the procedure detailed by Cao et al. [24] using the thiobarbituric acid reaction. Peel powder (0.2 g) was added to 2 mL of trichloroacetic acid solution and then centrifuged (10,000× *g*, 20 min) at 4 °C. The 0.2 mL supernatant was combined with 1 mL of 0.67% thiobarbituric acid, followed by boiling the mixture for 20 min and subsequent centrifugation after rapid cooling. Absorbance measurements were conducted on the ultimate supernatant at 532 nm, 450 nm, and 600 nm. These measurements were repeated thrice. The MDA content was calculated as follows:

$$\mathrm{MDA}\;\mathrm{content}\;(µ\mathrm{mol}/g\;\mathrm{FW})=\frac{c\times V}{Vs\times m\times 1000}$$

where *c* is the concentration of MDA in the reaction mixture (µmol/L), *V* is the volume of extraction buffer (mL), *Vs* is the volume of supernatant used for MDA detection (mL), and *m* is the mass of the Nanfeng tangerines (g).

### 2.8. Measurement of Fruit Peel Antioxidant-Related Characteristcs

Quantification of total phenol and flavonoid contents was performed in accordance with the methodologies specified by Zeng et al. [11]. 0.1 g of peel powder was dissolved in 4 mL a 1% ice-cold HCl–methanol solution. The supernatant, obtained post-centrifugation at 8000× *g* for 20 min at 4 °C, was utilized for absorbance measurements at 280 nm and 325 nm. The total phenol content was quantified as OD_280_/g FW, and the total flavonoid content was denoted as OD_325_/g FW.

Total antioxidant capacity was assessed following the protocol outlined by Zeng et al. [11]. Upon addition of 1.0 g of peel powder to 8 mL of anhydrous ethanol, the mixture was subjected to centrifugation at 12,000× *g* for 20 min at 4 °C. Obtaining the supernatant (0.1 mL), it was then mixed with 2.9 mL of a 2,2-diphenyl-1-picrylhydrazyl (DPPH) solution. After being kept in the dark for 30 min at room temperature, the reaction mixture was analyzed for absorbance at 517 nm. The DPPH free radical scavenging rate (%) was calculated by evaluating the total antioxidant capacity, and this measurement was repeated thrice.

The measurement of antioxidant enzymes peroxidase (POD), catalase (CAT), and superoxide dismutase (SOD) was conducted in accordance with the methodology described by Cao et al. [24], with some modifications. The POD (EC 1.11.1.7) activity was determined based on the oxidation of guaiacol. Dissolving 0.2 g of frozen peel in 2 mL of a 50 mmol/L acetic acid buffer (1 mM Polyethylene glycol, 4% Polyvinyl polypyrrolidone, and 1% tritonX-100, pH 5.5). After centrifugation (12,000× *g*, 30 min) at 4 °C, the supernatant (0.5 mL) was combined with 3 mL of 25 mM guaiacol and 0.2 mL of a 0.5 M H_2_O_2_ solution. The reaction mixture was employed for the detection of POD activity. One unit (U) of POD activity was recorded by monitoring a 1/min increase in absorption at 470 nm. The CAT (EC 1.11.1.6) activity was determined based on the decomposition of H_2_O_2_. In a 2 mL solution of 50 mM phosphate buffer (pH 7.5, with 5% Polyvinyl pyrrolidone and 5 mM Dithiothreitol), 0.2 g of peel powder was introduced. The supernatant (0.1 mL) was retrieved and combined with 2.9 mL of 20 mM H_2_O_2_ solution after centrifugation at 12,000× *g* for 30 min at 4 °C. The reaction mixture was employed for the detection of CAT activity. The definition of one U of CAT activity was the amount of enzyme required to decompose 1 µmol of H_2_O_2_ in 1 min under room temperature conditions. The SOD (EC 1.15.1.1) activity was determined based on its inhibitory effect on the reduction of nitroblue tetrazolium (NBT). The dissolution of 0.2 g of frozen peel powder in 2 mL of 100 mM extraction buffer (phosphate buffer containing 5% PVP and 5 mM DTT, pH 7.8) was performed. The supernatant, obtained after centrifugation (12,000× *g*, 30 min) at 4 °C, was used for SOD activity detection. The enzyme dosage required to inhibit 50% of NBT reduction at 560 nm was defined as one U of SOD activity. The POD, CAT, and SOD activities were quantified as U/g FW. All experiments were conducted in triplicate.

### 2.9. Data Analysis

The data are displayed as the mean ± standard error. Utilizing SPSS Statistics 25.0 (IBM, Chicago, IL, USA), data analysis was conducted using one-way analysis of variance. The analysis of mean variances involved the application of the least significant difference method and Duncan’s test, with statistical significance set at *p* < 0.05. Principal component analysis (PCA) was conducted through the ChiPlot analysis website (https://www.chiplot.online/)((accessed on 27th, November, 2024)), and PCA plots were generated and composite scores were calculated following the methodology outlined by Chen et al. [7]. Utilizing GraphPad Prism 8.0, the figures were constructed.

## 3. Results

### 3.1. Effect of Luteolin Treatment on Postharvest Quality of Nanfeng Tangerines

**Figure 2:**
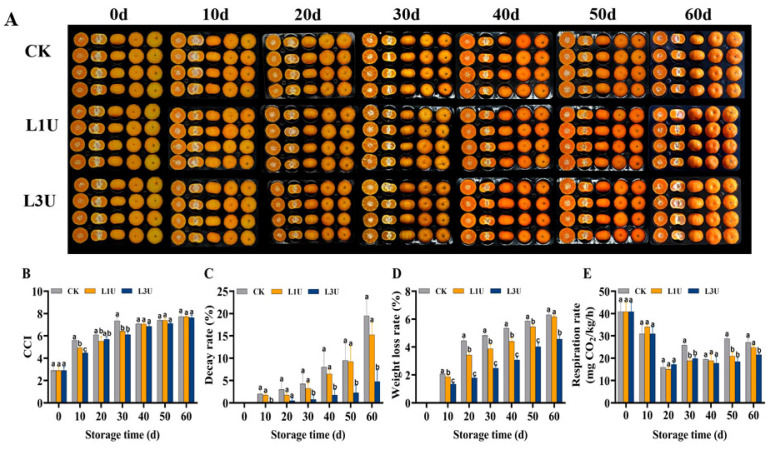
Effects of luteolin on postharvest quality of Nanfeng tangerines fruit when stored at room-temperature: (**A**) appearance, (**B**) citrus color index (CCI) value, (**C**) decay rate, (**D**) weight loss rate and (**E**) respiration rate. Vertical bars represent the standard error. Lowercase letters on a single day denoted a significant disparity (*p* < 0.05) between the control and luteolin treatment categories.

To evaluate postharvest quality changes under room-temperature storage following luteolin treatment, we examined the fruit peel color, decay rate, weight loss rate, and respiration rate. The peel color turns from greenish yellow to orange during Nanfeng tangerine room temperature storage (Figure 2A). The CCI value of the peels in the control group increased rapidly and then became relatively stable, whereas those in the luteolin treatment groups were maintained at a relatively low level (*p* < 0.05) during the first 30 d of storage (Figure 2B). As shown in Figure 2C, decay symptoms were observed in both untreated and luteolin-treated Nanfeng tangerines by Day 10 of storage, although luteolin treatment delayed the increase in decay. Moreover, the decay rate of fruits in the L1U and L3U treatment groups showed a significant reduction of 4.25% and 14.75%, respectively, compared to the control group after 60 d of storage (*p* < 0.05) (Figure 2C). Luteolin-treated fruits exhibited a consistently lower weight loss rate than untreated fruits throughout the storage period, and the L3U treatment dramatically reduced the weight loss (Figure 2D). As shown in Figure 2E, the respiration rate during storage was similar in the luteolin treatment groups, exhibiting an initial decline succeeded by a fluctuating rise. On Day 20, the luteolin treatment groups exhibited their lowest respiration rates and showed an inhibitory effect during the subsequent storage process when compared to the control group. These results indicate that treatment with 3 g/L of luteolin can effectively delay fruit senescence through suppressing fruit decay, weight loss, respiration rate, and color changes.

### 3.2. Effect of Luteolin on TSS, TA, and TSS/TA Ratio of Nanfeng Tangerines

**Figure 3:**
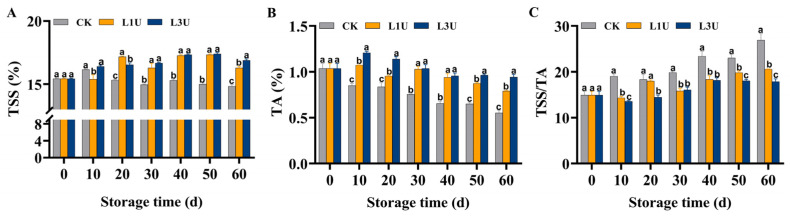
Effects of luteolin on: (**A**) total soluble solids (TSS), (**B**) titratable acid (TA), and (**C**) TSS/TA ratio of Nanfeng tangerines pulp during room temperature storage. Vertical bars represent the standard error of three replicates. Lowercase letters on a single day denoted a significant disparity (*p* < 0.05) between the control and luteolin treatment categories.

As shown in Figure 3A, the TSS content in the control pulp increased during the initial 10 days of storage; however, a marked decline was noted thereafter. The TSS content in the luteolin-treated fruit fluctuated and showed a significant increase (*p* < 0.05) after 10 d of storage when compared to that of untreated fruit. Over the storage period, the TA content exhibited a gradually decreasing pattern, with a significantly higher level (*p* < 0.05) observed in the pulp of both luteolin treatment groups compared to the control group (Figure 3B). As shown in Figure 3C, the TSS/TA ratio of the control pulp increased over the storage period. After luteolin treatment, the rate of increase in the ratio exhibited a statistically significant decrease in comparison to the control pulp (*p* < 0.05). These findings imply that treatment with luteolin effectively retards the decrease in sugar and acid content and the increase in sugar/acid ratio, thereby influencing fruit flavor quality.

### 3.3. Effect of Luteolin on Organic Acid Components of Nanfeng Tangerines

**Figure 4:**
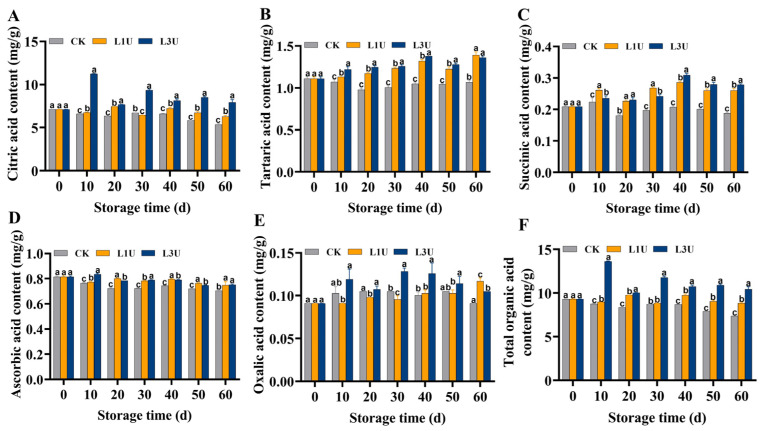
Effects of luteolin on: (**A**) citric acid, (**B**) tartaric acid, (**C**) succinic acid, (**D**) ascorbic acid, (**E**) oxalic acid, and (**F**) total organic acid contents in Nanfeng tangerines pulp during storage at room temperature. Vertical bars indicate standard error of three replicates. Lowercase letters on a single day denoted a significant disparity (*p* < 0.05) between the control and luteolin treatment categories.

Nanfeng tangerine organic acid components, namely citric acid, tartaric acid, succinic acid, oxalic acid, and ascorbic acid, were quantified and are shown in Figure 4. Citric acid represents a primary organic acid present in citrus fruits. The citric acid content decreased steadily during the storage period in the control group, whereas the decreasing trend was significantly retarded (*p* < 0.05) following luteolin treatment, especially in the L3U group (Figure 4A). The tartaric acid, succinic acid, and ascorbic acid contents in the control group exhibited a gradual decline, mirroring that of the citric acid content. Following luteolin treatment, the contents exhibited a significant increase compared to the control group throughout the storage period (*p* < 0.05) (Figure 4B–D). The oxalic acid level initially increased and then slowly decreased. The fruit treated with L3U exhibited a significantly higher oxalic acid content than the untreated fruit after 50 days of storage (Figure 4E). Citric acid constitutes the primary organic acid found in Nanfeng tangerines, and its degradation decreased the total organic acid content during storage at room temperature (Figure 4F). The luteolin-treated fruit exhibited a notably higher organic acid content compared to the untreated fruit, and L3U was more effective than L1U.

### 3.4. Effect of Luteolin on MDA and H_2_O_2_ Contents of Nanfeng Tangerines Peel

**Figure 5:**
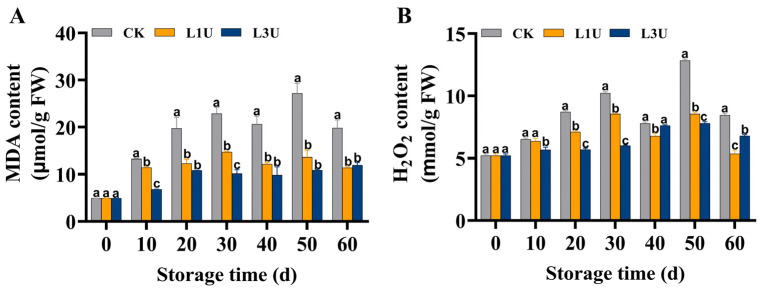
Examination of luteolin’s effects on: (**A**) malondialdehyde (MDA) content and (**B**) H_2_O_2_ content of Nanfeng tangerines peel during room temperature storage. Vertical bars indicate standard error of three replicates. Lowercase letters on a single day denoted a significant disparity (*p* < 0.05) between the control and luteolin treatment categories.

As depicted in Figure 4A, the MDA content in the peel of control fruit showed a fluctuating increase, succeeded by a rapid decrease on Day 60, whereas the MDA content in luteolin-treated fruit showed a mild increase throughout storage, significantly lower than in control fruit (*p* < 0.05) (Figure 5A). Similarly, a gradual increase in the H_2_O_2_ content was observed in the control group within 30 d of storage and fluctuated thereafter (Figure 5B). In the luteolin-treated groups, the H_2_O_2_ content showed a significant decrease in comparison to the control group (*p* < 0.05) (Figure 5B). In the three treatments, the MDA and H_2_O_2_ contents exhibited an increasing trend over time; however, the increasing intensity was significantly alleviated following L3U treatment (*p* < 0.05).

### 3.5. Effect of Luteolin on Total Phenol and Total Flavonoid Contents, Along with the DPPH Radical Scavenging Rate of Nanfeng Tangerines Peel

**Figure 6:**
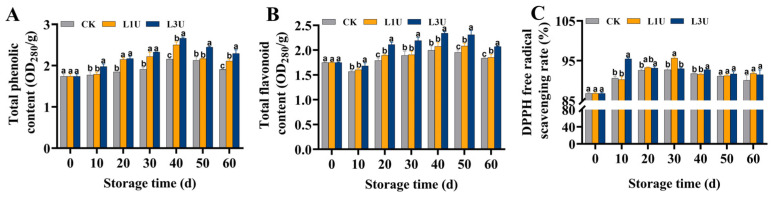
Impact of luteolin on: (**A**) total phenol content, (**B**) total flavonoid content, and (**C**) 2,2-diphenyl-1-picrylhydrazyl (DPPH) free radical scavenging rate of Nanfeng tangerines peel during room temperature storage. Vertical bars indicate standard error of three replicates. Lowercase letters on a single day denoted a significant disparity (*p* < 0.05) between the control and luteolin treatment categories.

As shown in Figure 6A,B, both untreated and luteolin-treated fruits showed a gradual increase in total phenol and flavonoid contents, with peak levels observed at 40 days of storage; however, a slight decrease in total flavonoid content was observed on Day 10. Luteolin treatment resulted in the maintenance of elevated total phenol and flavonoid contents (*p* < 0.05) compared to the control group. The DPPH free radical scavenging rate, which was evaluated as the total antioxidant capacity, exhibited a gradual rise over the initial 20 days of storage and then decreased (Figure 6C). Luteolin treatment markedly elevated (*p* < 0.05) the free radical scavenging rate after 30 d and 10 d of storage under L1U and L3U, respectively. These findings demonstrate that luteolin treatment boosts antioxidant capacity through the increase in total phenol and flavonoid contents. The effect of L3U treatment on the antioxidant capacity was greater than that of L1U treatment.

### 3.6. Effect of Luteolin on POD, CAT, and SOD Activities of Nanfeng Tangerines Peel

**Figure 7:**
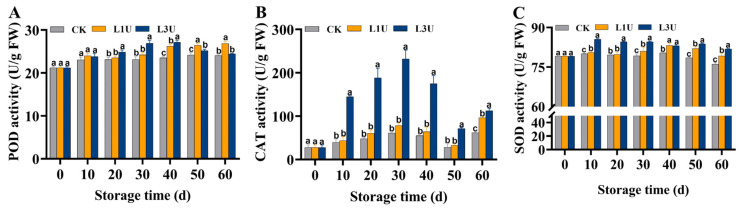
Effects of luteolin on: (**A**) peroxidase (POD), (**B**) catalase (CAT), and (**C**) superoxide dismutase (SOD) activities of Nanfeng tangerines peel during storage at room temperature. Vertical bars indicate standard error of three replicates. Lowercase letters on a single day denoted a significant disparity (*p* < 0.05) between the control and luteolin treatment categories.

As shown in Figure 7A, throughout the storage period, there was a mild increase in POD activity in the peels of the control group. Conversely, the POD activity in the peels of the L1U treatment group exhibited a rapid initial increase over the first 10 days, then experienced a mild decline and an increasing trend thereafter; however, it exhibited a notable increase in comparison to the control group (*p* < 0.05). Similarly, the L3U treatment group showed dramatically higher levels compared to the control group during Days 0–50 of storage. The CAT activity in the peels of the control and L1U treatment groups showed identical trends, with a mild rise during the initial 30 days of storage, followed by a mild decrease on Day 50, whereas the CAT activity in the L3U treatment group exhibited a substantial enhancement in comparison to the other two groups (*p* < 0.05) (Figure 7B). The SOD activity in the control fruit exhibited a gradual decrease during the initial 40 days of storage, and then rapidly decreased thereafter; nevertheless, the luteolin treatment groups exhibited a significant enhancement in activity (*p* < 0.05) (Figure 7C). Moreover, the SOD activity in L3U-treated fruit showed a notable increase than that in untreated fruit across the entire storage duration (*p* < 0.05) (Figure 7C). These results indicate that luteolin treatment enhanced the activity of antioxidant enzymes of POD, CAT, and SOD in Nanfeng tangerines during storage.

### 3.7. Effects of Luteolin on Storage Quality and Antioxidant Properties of Nanfeng Tangerines: PCA Analysis

**Figure 8:**
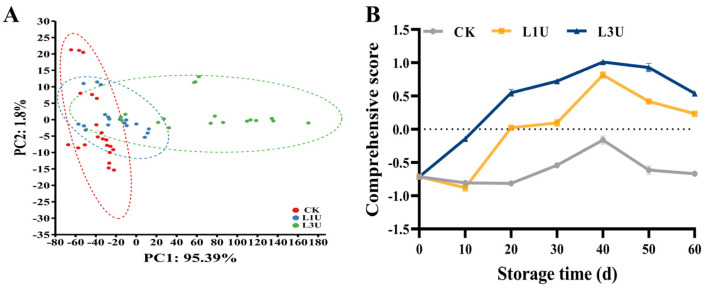
(**A**) Principal component analysis and (**B**) comprehensive score of Nanfeng tangerines treated with luteolin and stored at room temperatures.

Upon evaluating the quality and antioxidant attributes of Nanfeng tangerines, all data were subjected to PCA. The PCA results showed that the L3U-treated fruit was significantly differentiated from the control fruit via PC1 (95.39%) (Figure 8A). To ascertain the effectiveness of various concentrations of luteolin in maintaining the fruit quality and antioxidant capacity of Nanfeng tangerines, the comprehensive score was computed following the methodology outlined by Chen et al. [7]. As shown in Figure 8B, the composite scores of Nanfeng tangerines in the three groups increased gradually during the first 40 d and then decreased rapidly, whereas the score in untreated group was consistently negative. After 20 days of storage, the composite score in the luteolin treatment groups reached a positive value and significantly surpassed that of the control group. Moreover, the score under the L3U treatment was superior to that under the L1U treatment and peaked at 40 d, suggesting that L3U treatment may maintain better quality and antioxidant capacity of Nanfeng tangerines.

## 4. Discussion

### 4.1. Luteolin Treatment Delays Decay and Preserves Quality of Nanfeng Tangerines

Postharvest quality deterioration of citrus fruits occurs mainly because of high rates of weight loss, nutritional loss, disease, and subsequent decay during postharvest handling, all of which ultimately affect the market value [25]. Previous studies have reported that flavonoids (e.g., luteolin) are abundant in citrus fruits and have antioxidant and antimicrobial properties [17,26]. Moreover, luteolin solubility in ethanol is greater than 8 mg/mL [27], while in this study, it is around 10 mg/mL, fully meeting the experimental requirements. Luteolin has been proven to be effective in preserving the quality of fruits, notably sweet cherries [21] and winter jujubes [20], during storage. In the current investigation, similar results were observed, with significant delays in the decay and weight loss of Nanfeng tangerines following luteolin treatment, especially at a concentration of 3 g/L (Figure 2). The inhibition effect of luteolin on Nanfeng tangerine decay may be attributed to the antifungal efficacy against pathogens [21]. It is noteworthy that the decay and weight loss inhibition effect post luteolin treatment exceeded or equaled previous findings in Nanfeng tangerine studies. On day 60, the decay rate of luteolin treatment was 24.36% of CK, while the pre-harvest calcium-treated group was at 46.86% of CK [7], the nanoemulsion-treated group was at 44.59% of CK [9], and the SA-LL extract-treated group was at 20.09% of CK [10]. Similarly, the weight loss rate of CK was found to be 27.08% and 27.58% higher than that of the Calbit [7] and luteolin treatments, respectively.

In addition, the CCI values and respiration rates were lower in the luteolin treatment groups in comparison to the control group before and after 30 d of storage, respectively. In line with results reported in other studies, the respiration rate of the Calbit-treated group on day 30 was 78.86% of CK [7], whereas the luteolin-treated group showed a rate of 76.88% of CK. Furthermore, a marked reduction in CCI levels was noted in Nanfeng tangerines treated with SA-LL extract [10] and luteolin compared to the control group. These results suggest improved postharvest quality and enhanced disease resistance following luteolin application. However, much more extensive research is required to prove this promising potential for quality maintenance.

Furthermore, the solid/acid ratio, widely employed as the primary indicator of citrus internal quality at harvest maturity worldwide, determines citrus postharvest taste and flavor quality [28,29,30]. Our results showed that luteolin treatment led to higher TSS accumulation and delayed the decrease in TA, whereas the TSS/TA ratio was maintained at lower levels following luteolin treatment in comparison to the control group (Figure 3). These observed trends in the TSS/TA ratio were in line with those of coating-treated Nanfeng tangerines in a previous study [10]. Similar trends were also reported by Liu et al. [21], who demonstrated that treatment with 200 mg/L of luteolin could delay the decrease in TA content during the storage of sweet cherry at 25 °C. Taken collectively, these findings imply that luteolin effectively preserves the appearance and flavor quality in Nanfeng tangerines during storage at room temperature.

### 4.2. Luteolin Treatment Postpones Nanfeng Tangerine Organic Acid Degradation

Organic acids serve as crucial markers of fruit quality [31]. During fruit storage, the organic acid content affects fruit flavor, storage characteristics, and fruit senescence [32]. In citrus fruits, the deterioration of flavor quality in postharvest storage is mainly attributed to the degradation of organic acids, with citric acid being the most prevalent [22,28,33]. In this study, the citric acid content of Nanfeng tangerines decreased during room temperature storage, aligning with findings from Chen et al. [7]. In contrast, luteolin treatment maintained higher contents of citric acid and other organic acids, which was further confirmed by the delayed decrease in TA in Nanfeng tangerines (Figure 4A). This indicates that luteolin treatment could maintain a higher organic acid content and improved postharvest flavor quality in Nanfeng tangerines, showing promise as a method for maintaining fruit storage quality.

### 4.3. Luteolin Strengthens Nanfeng Tangerine Antioxidant Capacity

In this study, luteolin was observed to significantly delay the elevation of MDA and H_2_O_2_ contents, suggesting delayed senescence of Nanfeng tangerine fruit following luteolin application. Liu et al. [21] also reported that luteolin reduces the accumulation of MDA and ROS. As the main non-enzymatic antioxidant compounds, citrus phenols and flavonoids possess high antioxidant abilities, which serve as vital physiological mechanisms in the postponement of citrus fruit senescence and the maintenance of fruit quality [26]. Notably, total phenol and flavonoid contents were markedly enhanced following luteolin treatment (Figure 6). Furthermore, the improved DPPH free radical scavenging rate also suggested a significantly increased antioxidant capacity in the Nanfeng tangerine peel, which was further confirmed by the increased total phenol and flavonoid contents and decreased accumulation of MDA and H_2_O_2_ contents. These findings partially align with the impact of luteolin application on sweet cherry fruits [21]. Comparable outcomes were documented in winter jujube exposed to 200 mg/L luteolin [20], and Nanfeng tangerine treated with SA-LL extract [10] and 5 g/L naringin [11].

In addition to antioxidant compounds, fruit tissues can scavenge ROS by generating antioxidant enzymes, namely POD, CAT, and SOD [6,34,35]. In the current investigation, luteolin treatment led to enhancements in the enzymatic activities of POD, CAT, and SOD, accompanied by the inhibition of H_2_O_2_ content (Figure 7). These results were somewhat aligned with prior studies on sweet cherry and winter jujube, which showed that luteolin enhanced SOD, POD, and CAT activities [20,21]. Similar results were also documented in Nanfeng tangerine treated with nanoemulsion [9] and SA-LL extract [10]. These findings collectively verify that the increased antioxidant capacity associated with the enzymatic and non-enzymatic antioxidant systems triggered by luteolin generally led to ROS scavenging and quality maintenance.

## 5. Conclusions

In conclusion, our findings suggest that luteolin treatment significantly impacts the maintenance of Nanfeng tangerine postharvest quality and antioxidant capacity. Treatment with 3 g/L of luteolin markedly inhibited the acceleration of decay and weight loss rates, suppressed the respiration rate and color changes, and maintained TSS and TA contents and organic acid components at higher levels. Furthermore, luteolin treatment enhanced DPPH free radical scavenging by upregulating the levels of total phenols and flavonoids along with the antioxidant activities of POD, CAT, and SOD, and consequently, inhibiting the accumulation of MDA and H_2_O_2_. Overall, exogenous luteolin application to Nanfeng tangerines positively modulated postharvest fruit quality following enhanced antioxidant capacity, and this treatment exhibits great potential for maintaining fruit freshness during storage.

## Data Availability

The original contributions presented in the study are included in the article, further inquiries can be directed to the corresponding authors.
